# CSF complement 3 and factor H are staging biomarkers in Alzheimer’s disease

**DOI:** 10.1186/s40478-016-0277-8

**Published:** 2016-02-17

**Authors:** William T. Hu, Kelly D. Watts, Prashant Tailor, Trung P. Nguyen, Jennifer C. Howell, Raven C. Lee, Nicholas T. Seyfried, Marla Gearing, Chadwick M. Hales, Allan I. Levey, James J. Lah, Eva K. Lee, for the Alzheimer’s Disease Neuro-Imaging Initiative

**Affiliations:** Department of Neurology, Emory University School of Medicine, 615 Michael Street, 505 F, Atlanta, GA 30322 USA; Center for Neurodegenerative Diseases, Emory University School of Medicine, Atlanta, GA USA; Alzheimer’s Disease Research Center, Emory University School of Medicine, Atlanta, GA USA; Department of Biochemistry, Emory University School of Medicine, Atlanta, GA USA; School of Industrial and Systems Engineering, Georgia Institute of Technology, Atlanta, GA USA

**Keywords:** Amyloid beta, Diagnosis, C3, FH, Machine learning, Replication, Tau

## Abstract

**Introduction:**

CSF levels of established Alzheimer’s disease (AD) biomarkers remain stable despite disease progression, and non-amyloid non-tau biomarkers have the potential of informing disease stage and progression. We previously identified complement 3 (C3) to be decreased in AD dementia, but this change was not found by others in earlier AD stages. We hypothesized that levels of C3 and associated factor H (FH) can potentially distinguish between mild cognitive impairment (MCI) and dementia stages of AD, but we also found their levels to be influenced by age and disease status.

**Results:**

We developed a biochemical/bioinformatics pipeline to optimize the handling of complex interactions between variables in validating biochemical markers of disease. We used data from the Alzheimer’s Disease Neuro-imaging Initiative (ADNI, *n* = 230) to build parallel machine learning models, and objectively tested the models in a test cohort (*n* = 73) of MCI and mild AD patients independently recruited from Emory University. Whereas models incorporating age, gender, APOE ε4 status, and CSF amyloid and tau levels failed to reliably distinguish between MCI and mild AD in ADNI, introduction of CSF C3 and FH levels reproducibly improved the distinction between the two AD stages in ADNI (*p* < 0.05) and the Emory cohort (*p* = 0.014). Within each AD stage, the final model also distinguished between fast vs. slower decliners (*p* < 0.001 for MCI, *p* = 0.007 for mild AD), with lower C3 and FH levels associated with more advanced disease and faster progression.

**Conclusions:**

We propose that CSF C3 and FH alterations may reflect stage-associated biomarker changes in AD, and can complement clinician diagnosis in diagnosing and staging AD using the publically available ADNI database as reference.

**Electronic supplementary material:**

The online version of this article (doi:10.1186/s40478-016-0277-8) contains supplementary material, which is available to authorized users.

## Introduction

Diagnosis for Alzheimer’s disease (AD) is significantly enhanced by the introduction of objective etiologic biomarkers, [1–3] but there is currently no fluid or imaging marker to provide unbiased staging information to complement clinician judgement. Importantly, levels of cerebrospinal fluid (CSF) AD biomarkers, including beta-amyloid 1–42 (Aβ42), total tau (t-Tau), and tau phosphorylated at threonine 181 (p-Tau_181_), remain relatively stable after disease onset, and do not differentiate between mild cognitive impairment due to AD (MCI) and mild AD dementia [4, 5]. In addition to amyloid and tau biomarkers, the CSF proteome is a ready source of non-amyloid, non-tau (NANT) biomarkers. These CSF proteins can provide staging information related to the degree of neurodegeneration or secondary events associated with different disease stages, but successful replication of NANT biomarkers for AD has been hampered by differences in cohorts, platforms, and analyses to allow for clinical translation.

Among candidate NANT biomarkers, we previously found CSF C3 levels to be altered in AD using a commercial immunoassay panel [6]. These results were replicated in one study using demented AD patients [7] but not another using non-demented patients from earlier stages (Clinical Dementia Rating of 0.5) [8]. Discrepant findings have also been reported for CSF C3 and its interacting partner factor H (FH) by mass spectrometry-based methods [9–11]. Several lines of evidence point to the importance of characterizing complement activation through biomarkers in AD. Genome wide association studies have shown polymorphisms in complement receptor 1 (*CR1*) to be associated with genetic risks to AD [12]. Multiple components of the classical complement pathways have been associated with neuritic plaques and cerebrovascular amyloid [13], and mouse models for AD with reduced classical pathway activation showed reduced neuropathology [14]. As biomarker-driven clinical trials gain traction in AD, it is critical to identify AD patients with and without classical complement activation at baseline. Because discrepant findings related to C3 and FH resulted from preferential analysis of early (mild cognitive impairment, MCI) or late (dementia) AD stages, we hypothesized that C3 and FH levels were altered (in keeping with complement activation) during the transition from MCI to dementia in AD. At the same time, cut-off values may be difficult to derive because demographic variables (such as age-related changes) and comorbid conditions can confound the interpretation of C3 and FH. This limitation can be overcome with machine learning (ML) strategies built on real world data similarly confounded by complex interactions to predict class membership, but often suffer from over-training which limits their generalization [6, 8].

**Fig. 1:**
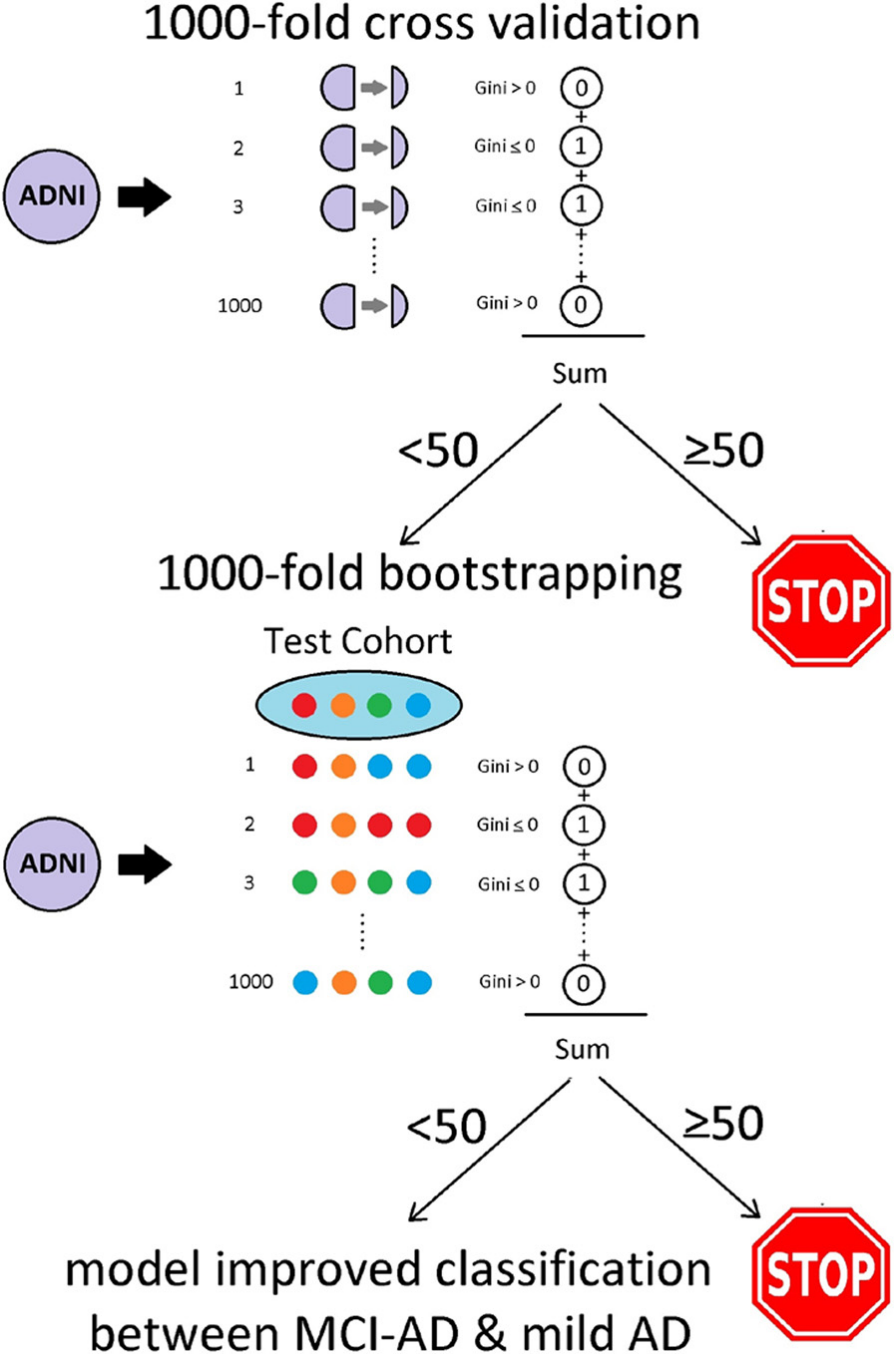
Graphical representation of XMITTN. Two independent datasets were included, with the ADNI cohort as the training set and the Emory cohort as the independent test set. Within the ADNI cohort, 1000-fold cross validation is performed with each biomarker feature set (without or without C3 and FH) to determine which biomarker-ML combination results in internally validated separation between MCI and AD. The successful biomarker-ML combination is then tested in the test set through 1000-fold bootstrapping

Here we built a new analytical pipeline to bridge the gaps between biomarker development, independent cohort testing, machine learning, and t-statistics to determine if CSF C3 and FH are useful staging biomarkers in AD. This method, which we call XMITTN (cross[X]-validation, Machine learning, Independent Training and Test set, and Null hypothesis testing, Fig. 1), objectively assesses nine ML algorithms in any given dataset through 1000-fold cross-validation in a training dataset, and chooses appropriate ML models according to *a priori p*-values. The learned biomarker-ML models – not just the biomarker levels – are then directly applied to the test dataset without building new models, with *p*-values generated through 1000-fold bootstrapping. We used data from the publically available multi-center Alzheimer’s Disease Neuro-imaging Initiative (ADNI) and an independent cohort at Emory University to test our hypothesis that a biomarker-ML combination provides staging information in AD.

## Methods

### Study participants

Two cohorts of patients were included in the current study. ADNI data used in the preparation of this article were obtained from the ADNI database (adni.loni.ucla.edu; adni.loni.usc.edu). Briefly, ADNI (PI: Michael W. Weiner, MD) is the result of efforts of many co-investigators from a broad range of academic institutions and private corporations, and subjects have been recruited from over 50 sites across the U.S. and Canada [15]. The initial goal of ADNI was to recruit 800 adults, ages 55 to 90, to participate in the research, approximately 200 cognitively normal older individuals to be followed for three years, 400 people with MCI to be followed for three years and 200 people with early AD to be followed for two years (www.adni-info.org). ADNI-1 enrolled about 800 participants with multiple longitudinal biomarker and cognitive measurements at 6 or 12 month intervals up to four years.

The Emory validation cohort included 73 consecutive patients recruited and longitudinally followed in the Emory Cognitive Neurology Clinic or the Emory Alzheimer’s Disease Research Center. The study was approved by the Emory University Institutional Review Board, and informed consent was obtained from all subjects or their authorized representatives. All participants underwent standard neurological and cognitive assessments and were assigned diagnosis according to consensus criteria including those for mild cognitive impairment (MCI) [16, 17] and AD (Clinical Dementia Rating 1 or 2) [18, 19]. For the purpose of this study, only MCI subjects with CSF biomarkers consistent with AD were included (*n* = 51). Compared to ADNI subjects, Emory subjects were younger (68.7 vs. 74.5 yr, *p* < 0.001), more likely to be women (53.4 % vs. 39.6 %, *p* = 0.037), and less likely to have the APOE ε4 allele (45.2 % vs. 66.5 %, *p* = 0.001, Table 1).

**Table 1:** Demographic and biomarker information for subjects from Emory and ADNI

	ADNI		Emory	
	MCI (*n* = 135)	AD (*n* = 95)	MCI (*n* = 51)	AD (*n* = 22)
Male (%)	86 (64 %)	53 (56 %)	28 (55 %)	6 (27 %)
Age (S.D.), yr	74.7 (7.6)	74.3 (7.7)	69.0 (7.4)	65.6 (8.8)
Education (S.D.), yr	15.8 (2.9)	14.9 (3.1)	15.4 (2.5)	13.9 (2.4)
Having at least one APOE4 allele	86 (64 %)	67 (70 %)	25 (49 %)	8 (36 %)
CSF				
Aβ42 (pg/mL)	136.7 (31.4)	143.5 (39.9)	129.8 (55.3)	168.3 (104.0)
t-Tau (pg/mL)	122.8 (60.6)	122.5 (57.8)	97.7 (49.6)	120.3 (60.0)
p-Tau_181_ (pg/mL)	42.4 (16.1)	41.4 (19.9)	55.6 (25.7)	61.1 (26.2)
FH (pg/mL)	1568 (629)	1750 (835)	1594 (493)	1692 (474)
Z-score, log(C3)	−0.060 (0.929)	0.061 (1.084)	−0.481 (1.031)	−0.064 (0.784)

MCI: mild cognitive impairment with CSF t-Tau/Aβ42 ≥ 0.39; AD: mild dementia due to Alzheimer's disease

### Procedures

CSF samples from ADNI subjects were collected as previously described [20, 21]. Samples from Emory subjects were collected according to strict protocols. At collection, participants were ≥21 years of age and in good general health, having no other psychiatric or major medical diagnoses that could contribute significantly to cognitive impairment or dementia other than the primary neurodegenerative disorder. CSF samples were collected between 8 AM and 2 PM without overnight fasting. These time frames were chosen as CSF Aβ42 levels during these times represent approximately 95 %-105 % of average CSF Aβ42 over time [22]. CSF was immediately aliquotted after collection and before freezing, and otherwise we used the ADNI biofluid protocols including the use of 24 G Sprotte needles, aspiration syringes, and transfer into 15 mL polypropylene tubes.

#### Biomarker measurement in ADNI

CSF levels of Aβ42, t-Tau, and p-Tau_181_ in ADNI were measured as previously described. MCI subjects with CSF Tau/Aβ42 ratio greater than or equal to 0.39 were classified as having MCI due to AD (abbreviated as MCI hereafter). CSF levels of FH and C3 were measured according to a modified manufacturer's protocol [23]. Briefly, 4 μL of never-thawed CSF was diluted at 1:2 with a protease inhibitor mix, and then further diluted a final dilution of 1:800. 50 μL of the diluted CSF was mixed with 25 μL of beads and 25 μL of buffer and allowed to mix on shaker for 18 hr at 4 °C (final CSF dilution 1:1600). After thorough washing, 25 μL of secondary antibodies were added to the beads and allowed to mix for 3 hr at room temperature. Substrates were allowed to develop for 30 min at room temperature. CSF C3 and FH levels were available for 135 subjects with MCI (CSF t-Tau/Ab42 ≥ 0.39) and 95 subjects with mild AD. These 230 subjects were entered for subsequent analyses.

#### Biomarker measurement in the independent validation cohort

CSF AD biomarker levels (Aβ42, t-Tau, p-Tau_181_) among Emory subjects were measured using the commercially available INNO-BIA AlzBio3 kits (Fujiribio, Ghent, Belgium). Our center achieves an average inter-plate coefficient of variation of 11.2 % for Aβ42, 10.2 % for t-Tau, and 13.8 % for p-Tau_181_. Only MCI subjects with CSF Tau/Aβ42 ratio greater than or equal to 0.39 were included. CSF C3 and FH levels were measured using the commercially available Milliplex MAP Kit: Human Neurodegenerative Disease Panel (Millipore, Billerica, MA) in the xMAP Luminex platform (Luminex Corp, Austin, TX) using a modified manufacturer's protocol. Specifically, 5 μL of never-thawed CSF was diluted at 1:400 without protease inhibitors, and 25 μL of CSF was added to a mixture containing 25 μL of antibody-beads and 50 μL of buffer (to give sufficient volume for mixing) for 2 hr at room temperature (final CSF dilution of 1:1600). After thorough washing, 25 μL of secondary antibodies were then added to the beads and allowed to incubate for 1 hr at room temperature. Substrates were allowed to develop for 30 min at room temperature. The lower limit of detection was 143 pg/mL for C3 and 7 pg/mL for FH. Our center achieves an average inter-plate coefficient of variation of 8.8 % for C3 and 7.8 % for FH, and individual samples with >20 % variations in measurements were repeated. A subset of samples (8 MCI and 8 mild AD) were also analyzed by liquid chromatography-tandem mass spectrometry (LC-MS/MS) to account for potential technical differences in measurements.

#### CSF C3 and FH levels - Mass Spectrometry

CSF C3 and FH levels were also analyzed in 16 randomly chosen Emory MCI and mild AD subjects in two independent runs of (LC/MS-MS) as described previously [24]. Aliquots of CSF (20 μL) from each subject was resolved on a 10 % SDS-PAGE gel and then five gel bands corresponding to molecular weight ranges underwent overnight trypsin digestion. Extracted peptides were analyzed by LC-MS/MS on a hybrid LTQ XL Orbitrap mass spectrometer (ThermoScientific). The MS/MS spectra were then matched to a complete semi-tryptic human protein database (NCBI reference database v.54) utilizing a target-decoy approach and peptides spectral matches filtered until achieving a false discovery rate (FDR) of < 1 % [25, 26]. Label free relative protein quantification was performed based on peptide spectral counts (SCs) and the extracted ion current measurements. Mann Whitney U-tests were used to compare FH and C3 levels between MCI-AD and mild AD (*n* = 16) due to the small sample size.

### Statistical analysis

Baseline statistical analyses and longitudinal cognitive analyses were performed using IBM SPSS Statistics Version 22 (IBM, Armock, NY). At baseline, chi-squared tests were used to determine differences in dichotomous variables, and Student’s T-tests or analyses of variance were used to analyze continuous variables. CSF C3 levels were not distributed normally and were log-transformed. Due to differences in Emory and ADNI protocols in primary incubation time and temperature (ADNI assay no longer available), C3 levels measured from Emory subjects were lower than C3 levels from ADNI subjects (no significant difference in FH levels). For comparison purposes, log-transformed C3 levels were normalized into Z-scores using C3 levels from cognitively normal subjects in ADNI (*n* = 115) or Emory (*n* = 25, Additional file 1: Table S1). FH levels were distributed normally and not adjusted before analysis.

Nine ML algorithms were included in XMITTN: logistic regression, perceptron, decision tree, boosted decision tree, gradient boosting, Naïve Bayesian, random forests (RF), K-nearest neighbors, and support vector machine (SVM). Some of these were previously applied to CSF AD biomarkers analysis [6, 8], but the importance of each biomarker can be over-stated due to these algorithms’ intrinsic properties and over-training. We designed XMITTN to evaluate these models through null hypothesis testing in four stages:

Stage One involves 1000-fold cross validation (CV) using the training (ADNI) cohort. 1000 random chosen training cohorts (*n* = 180) are simultaneously processed through each of the 9 ML algorithms, with 1000 matching sets of internal CV cohort (*n* = 50). In Stage Two, each ML will perform two classification experiments on the same training/CV cohorts. Experiment 1 incorporated 6 features: age, gender, presence of at least one APOE ε4 allele, CSF Aβ42, CSF t-Tau, and CSF p-Tau_181_. Experiment 2 had all features included in Experiment 1 plus levels of C3 and FH. Within each training cohort x ML algorithm x experiment combination, a model is built to maximize the classification between MCI and mild AD, and then tested on the CV cohort. The performance of each unique model generates a Gini index (from 0 to 1) which assesses the improvement in classification performance from chance alone, such that every ML algorithm will have an associated Gini index for each CV cohort. Because of the random separation of the ADNI cohort into 1000 training and CV sets, the Gini indices over 1000-fold CV form a normal distribution curve for each ML algorithm x experiment combination.

In Stage Three, the performance of each ML algorithm will be assessed by null hypothesis testing in the CV cohorts using the distribution curve of Gini indices. In this stage, the null hypothesis is true when the classification by ML is no better than chance alone. Because classification accuracy by chance alone has Gini index of 0, the frequency of this across the 1000-fold CV is equivalent to the area under the curve to the left of Gini index of 0. When the Gini index cannot be logically less than zero (classification performance is equivalent to chance when Gini index < 0), the frequency of Gini = 0 for each ML algorithm x experiment combination is equivalent to the *p*-value for null hypothesis testing. This allows for a more objective selection of ML algorithms for the independent test cohort, with the threshold of *p* < 0.05 in Experiment 1 or 2 across 1000-fold CV for each ML algorithm. Because each model is to be tested in an independent validation cohort, we did not adjust for multiple comparisons at this stage.

The final stage of XMITTN applies the model(s) optimized on the training (ADNI) set to the independent Emory test set. Conventionally, one accuracy measure will be calculated from the test set using a cut-off value derived from the training set. Applying the principle of null hypothesis testing to the test set, we used bootstrapping to generate 1000 evenly distributed test sets (50 MCI-AD, 50 AD). Gini indices derived from these sets can then be compared with classification by chance alone, with the frequency of Gini = 0 being equivalent to the *p*-value. If this final *p*-value is less than 0.05 or threshold adjusted for multiple comparisons, then the particular experiment (feature set x ML algorithm combination) was interpreted as demonstrating a significant improvement from classification by chance alone in two separate datasets.

## Results

### Univariate analysis of CSF C3 and FH levels in two datasets and two platforms

**Fig. 2:**
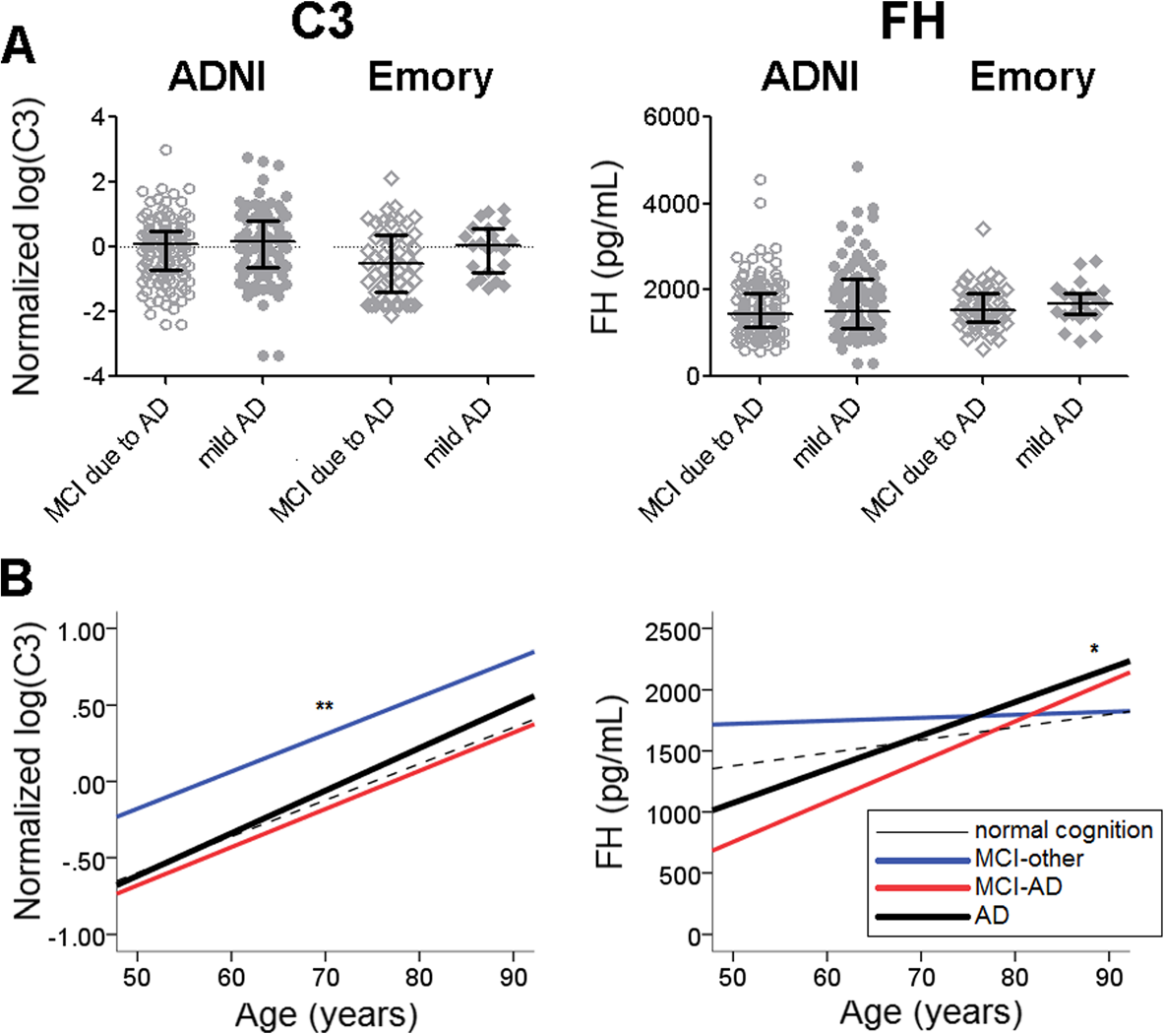
CSF C3 and FH levels according to diagnosis in the ADNI and Emory cohorts. MCI subjects only include those whose CSF t-Tau/Aβ42 ratio is greater than 0.39. Bars represent median values with interquartile range. Univariate analyses did not show any difference in C3 and FH levels between MCI and AD (panel **a**), but biomarker levels were strongly influenced by age (panel **b**; *p* < 0.001 for C3, *p* = 0.001 for FH). MCI-Other: initial clinical diagnosis with CSF t-Tau/Aβ42 < 0.39

We found no significant differences in CSF levels of C3 and FH between MCI and mild AD in the training or test set (Fig. 2). A subset of subjects (8 MCI and 8 mild AD) were analyzed by LC/MS-MS which also failed to show any significant level differences (data not shown), suggesting that the findings were not biased by the platform.

### CSF C3 and FH levels are influenced by many factors

At the same time, it is not surprising that direct comparisons of biomarker levels without taking into account influences of age, gender, and other factors would show no level differences. To better understand if CSF C3 and FH levels are influenced by demographic factors, we performed mixed linear analysis in the entire ADNI CSF dataset (including those with normal cognition and MCI-Other, Additional file 1: Table S2). For C3, there were main effects from age, diagnosis, and Aβ42, with older subjects and MCI-Other subjects having greater CSF C3 levels. For FH, there were main effects from age, diagnosis, diagnosis x age, Aβ42, and p-Tau_181_. Older patients with CSF consistent with AD have higher FH levels, but age did not influence FH levels among subjects whose CSF AD biomarkers were normal. Thus, even though univariate analysis did not reveal any CSF C3 and FH level differences between MCI and mild AD, direct comparison of C3 and FH levels without accounting for effects from age and diagnosis at the patient and biomarker level – rather than at the model level – may have masked stage-dependent differences in C3 and FH levels. Approaches beyond univariate analyses are thus necessary to address whether C3 and FH levels differed between MCI and mild AD.

### Using machine learning to analyze CSF C3 and FH levels in MCI and mild AD

**Fig. 3:**
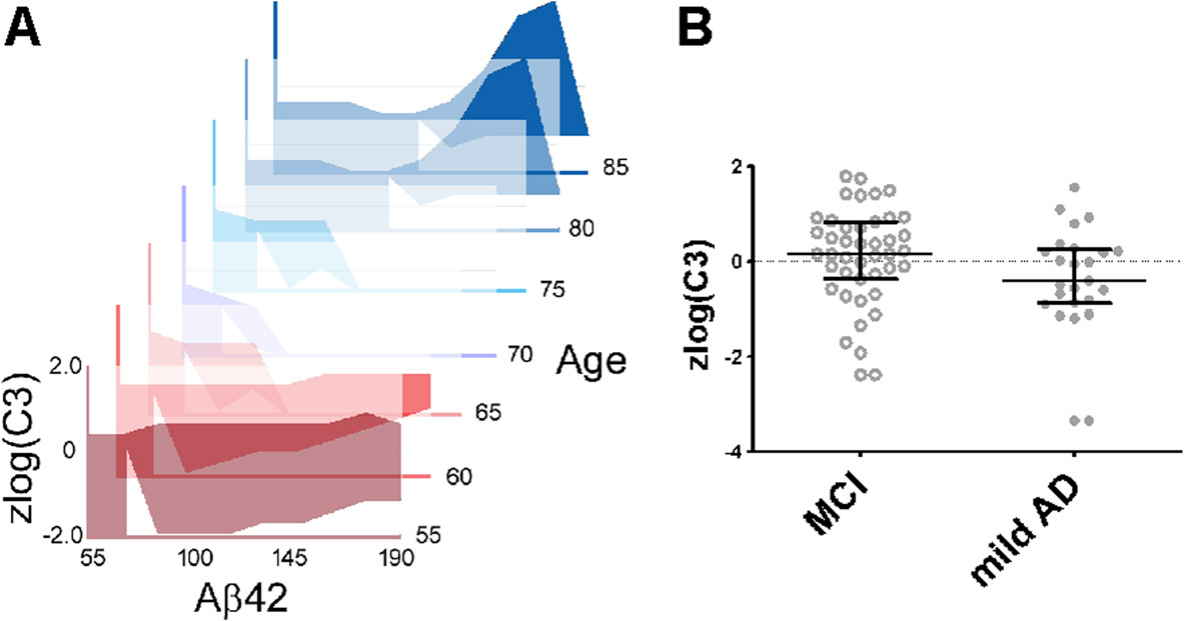
Hyperplanes separating MCI and mild AD according to XMITTN according to age, Aβ42, and C3 in men with APOE ε4 allele. Using ADNI data, XMITTN constructed a high-dimensional space to model the interaction between biomarkers and diagnosis. To visualize the space, we assigned fixed values to CSF t-Tau and FH, and plotted the planes which distinguishes between MCI and mild AD according to age (depth), CSF Aβ42 levels (X-axis), and zlog(CSF C3) (Y-axis; panel **a**). Panel A represents a model of C3 in MCI-AD and mild AD with matching gender (male), age (Y-axis), t-Tau, and FH. Subjects whose biomarker combinations fall into the filled regions are considered to have mild AD, and subjects whose biomarker combinations into the empty regions are considered to have MCI. Examining zlog(C3) levels between MCI and mild AD subjects with CSF < 125 pg/mL showed trend detected by XMITTN (panel **b**, *p* = 0.07)

To account for the complicated relationship between C3, FH, age, diagnosis, and established CSF AD biomarkers, we used XMITTN to determine if the introduction of C3 and FH levels into a two-class classification model can enhance the distinction between MCI and mild AD. In the ADNI cohort, we found through 1000-fold CV that, as expected, a biomarker panel including demographic variables, APOE ε4 status, and CSF amyloid and tau biomarkers could not sufficiently distinguish between MCI and mild AD using any of the nine ML algorithms (Table 2). After we introduced C3 and FH as additional features, we found improved classification of MCI and mild AD in two algorithms (*p* = 0.043 for RF, *p* = 0.033 for SVM). We then applied the RF and SVM algorithms built on the training dataset to the independent test dataset. The ADNI SVM model (Fig. 3) reproducibly distinguished between MCI and mild AD in the Emory cohort (*p* = 0.014) but the ADNI RF model did not (*p* = 0.595). In the Emory cohort, the ADNI SVM model had an average sensitivity of 59.3 %, an average specificity of 62.9 %, and an average accuracy of 61.1 % over 1000 bootstrapped samples.

**Table 2:** XMITTN output for ADNI and Emory cohorts assessing whether classification using two sets of variables is better than chance

Machine learning algorithm	*p*-value, Experiment 1, ADNI cohort	*p*-value, Experiment 2, ADNI cohort	*p*-value, Experiment 1, Emory cohort	*p*-value, Experiment 2, Emory cohort
Logistic	0.510	0.140		
Perceptron	0.792	0.912		
Decision Tree	0.197	0.161		
Random Forests	0.128	**0.043**	0.560	0.595
Naïve Bayes	0.403	0.367		
K-Nearest Neighbor	0.106	0.069		
Boosted Decision Tree	0.399	0.245		
Gradient Boosting	0.185	0.104		
Support Vector Machine	0.125	**0.033**	0.266	**0.014**

Experiment 1 includes 6 features: age, gender, presence of at least one APOE ε4 allele, CSF Aβ42, CSF t-Tau, and CSF p-Tau_181_, and no ML algorithm performed better than chance in distinguishing between the two AD stages. Experiment 2 has all previous features plus C3 and FH and levels, and achieved improved classification in two algorithms in the ADNI cohort and support vector machine in the Emory cohort (p < 0.05 shown in bold)

### Association between biomarker-based staging and cognitive functions

**Fig. 4:**
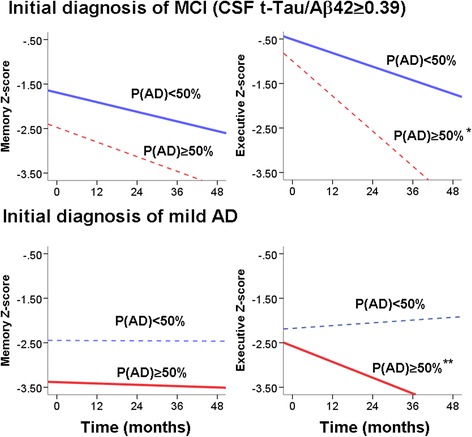
Longitudinal cognitive profiles of subjects in the test set according to initial clinical diagnosis and XMITTN-derived re-classification. P(AD) is the probability of subjects having mild dementia. MCI patients with P(AD) ≥ 50 % have greater executive dysfunction than MCI patients with P(AD) < 50 % (*p = 0.001) and a trend of greater memory dysfunction (*p* = 0.078). Mild AD patients with P(AD) ≥ 50 % have greater rates of decline in executive functions than mild AD patients with P(AD) < 50 % (**p = 0.007)

Because CSF amyloid and tau biomarker levels are associated with differences in longitudinal cognitive functions, we hypothesized that classification using CSF C3 and FH levels can enhance the prediction of longitudinal cognitive decline. For each subject in the Emory cohort, we calculated a dementia probability score P(AD). A subject who is consistently classified as mild AD in combined ADNI-Emory SVM validations has a P(AD) of 100 %, and a subject who is consistently classified as MCI across all SVM validations has a P(AD) of 0 %. For simplicity, we divided MCI subjects in the Emory cohort into those with P(AD) < 50 % (likely MCI) and those with P(AD) ≥ 50 % (likely mild dementia). Among MCI subjects with longitudinal follow-up (*n* = 44), the P(AD) < 50 % group had less executive dysfunctions than P(AD) ≥ 50 % group (difference in Z-score of 0.48, 95 % CI 0.20 – 0.77, *p* = 0.001, Fig. 3), as well as a trend of less memory dysfunction (difference in Z-score of 0.78, 95 % CI −0.09 – 1.66, *p* = 0.078, Additional file 1: Table S3). We interpret this as the P(AD) ≥ 50 % MCI group having more severe cognitive dysfunction and a cognitive phenotype closer to mild AD. Among mild AD subjects with longitudinal follow-up (*n* = 10), the P(AD) < 50 % group had slower rates of decline in executive functions, with a difference in Z-score of 0.035 per month (95 % CI 0.011 – 0.059, *p* = 0.007, Fig. 4). There was also a trend that the P(AD) < 50 % having less memory dysfunction than the P(AD) ≥ 50 % group. We interpret this as the P(AD) < 50 % mild AD group having less severe cognitive dysfunction and a cognitive phenotype closer to MCI. Thus, using longitudinal cognitive data and XMITTN, we confirmed that the ADNI SVM model incorporating C3 and FH levels can enhance the distinction between MCI and mild AD in a completely different patient population.

## Discussion

C3 and FH levels have been repeatedly associated with AD in the past, although the findings were not always consistent between studies. We analyzed the complex relationship between C3, FH, established CSF AD biomarkers, and AD stages, and developed a statistically rigorous analytical pipeline XMITTN to select not only demographic and fluid biomarkers but also ML algorithms themselves as biomarkers. Using clinical diagnosis as pseudo-gold standard, we successfully improved the distinction between early (MCI) and later (mild dementia) stages of AD in the independent (ADNI) and test (Emory) datasets. Importantly, the XMITTN-driven subclassification according to P(AD) within each clinical AD stage identified endophenotypes with different patterns of cognitive impairment, especially involving the executive domain. We propose that a biochemical model predicting AD stages built on a publically available dataset (ADNI) will enhance individual clinicians’ diagnostic assignment of MCI vs. AD in clinical and research settings.

Replication of initially promising results remains a fundamental challenge in translational biomarker research [27]. Our hypothesis related to C3 being a potential classifier between MCI and mild AD stemmed from conflicting findings of previous studies using CSF immunoassays [6–8, 28]. One of us (WTH) found CSF C3 levels to be decreased in mild AD in an autopsy-confirmed series [6], but a follow-up study using the same commercial analytical platform showed subjects with Clinical Dementia Rating of 0.5 had normal CSF C3 levels [8]. A recent analysis using ADNI data also showed minimal difference in absolute C3 and FH levels, even though there was a hint that C3 and FH levels were associated with cognitive decline over time [23]. While there have been significant discussions on the standardization of CSF collection and assay performance, there is little uniformity in which statistical or informatics-based approach should be used to analyze biomarker measures. Linear regression remains the most common first pass strategy to account for factors known to influence biomarker levels, and ML approaches are preferred to account for influences from known and unknown factors. At the same time, ML algorithms are designed to optimize desired outcomes in a single cohort, which makes it difficult to assess whether the ML-outcome pairing is reproducible. Such problems are not unique to fluid biomarker analysis, as MRI data are often subjected to various analytical platforms which sometimes give rise to incongruent conclusions. It is thus critical to recognize that the translation of any promising biomarker to clinical applications involves not only assay standardization but also analytical standardization. In other word, the selection of the appropriate model and training set is likely as important as the biomarker identities. As others have pointed out [29], the exact context of biomarkers (screening in a community cohort, differential diagnosis, clinical trial design) also needs to be considered in the construction of future ML models such that the appropriate model is as reliable and reproducible as the actual biochemical analytes themselves [30].

Exactly how FH and C3 alterations are mechanistically involved in AD remains unclear. C3 and FH polymorphisms have been both associated with AD [31]. Earlier data showed that C3 deactivation enhanced AD pathology in transgenic mice [32], and more recent data showed that acetylcholine enhances C3 activation in astrocytes *in vitro* [33]. FH regulates C3 levels [34], is itself found on neuritic plaques [35], and may be regulated by amyloid toxicity [36]. CSF C3 and FH levels may thus directly reflect the interplay between AD neuropathology and neuroinflammation. At the same time, altered CSF levels of C3 and FH may not be unique to AD, as similar changes have been reported in frontotemporal degeneration [6] and α-synucleinopathies [7]. The XMITTN model suggests that mild AD is associated with lower C3 levels than MCI, and it is likely that the imperfect syndromic diagnosis is not the ideal gold standard for model building. Instead of considering MCI and AD as two distinct entities each with a homogeneous composition, the biggest advantage of model like XMITTN may be its ability to identify distinct subgroups, each with relatively uniform membership according to objective biomarkers. Future studies can determine if patients showing CSF complement changes continue to demonstrate similar biochemical changes longitudinally, and whether there is a transition to cell-mediated inflammation in later AD stages. Treatment trials can also optimally match the therapeutics’ proposed mechanism of action with the matching patients to maximize the chance of a positive outcome.

Like the analytes and interactions they are designed to model, not all MLs have the same theoretical underpinning or application. The performance of each ML is dependent on sample size, data dimensionality, and inter-relationship between the variables. For example, logistic regression relies on the assumption that there is sufficient power to model the association between independent and dependent variables as linear relationships. Tree-based approaches such as RF and gradient boosting do not rely on linear assumptions, and can better handle categorical independent variables. Naïve Bayesian performs better in the absence of interactions between independent variables, and do not project well onto a CV or test set when a particular feature combination is not present (probability of 0) in the training set. SVM using a linear kernel is similar to logistic regression, but using a non-linear kernel creates more freedom in modeling with better handling of high dimensional data and noise. At the same time, better performance and over-training often come hand-in-hand [37, 38]. While computational methods exist to reduce overtraining [39], replication of the model (including biomarker levels and ML algorithm) across independent cohorts should remain the gold standard in identifying reproducible findings [40].

This study is built on two large, independent, well-characterized cohorts and detailed biochemical and bioinformatics analysis, but also has some weaknesses. CSF C3 levels were measured at Emory and ADNI using different assays due to the discontinuation of ADNI C3 assays, but the Z-score transformation within each cohort and the use of a high-dimensional data analysis allowed comparison of the two cohorts' data. We did not examine the conversion rate from MCI to dementia, as the clinician-based assessment of conversion is susceptible to the same bias as the distinction between MCI and dementia. Because our hypothesis bore from previous studies on MCI-AD and mild AD dementia, we did not assess our model as a classifier between pre-symptomatic AD and MCI-AD, or between mild and severe AD dementia which can be assessed in the future. Since studies have failed to show any level difference between MCI-AD and healthy seniors, the likelihood of a C3/FH model distinguishing between mild and severe AD dementia is much greater than it identifying MCI-AD from normal aging. We also did not incorporate findings from cerebral amyloid imaging, as an independent ML-based assessment of cerebral amyloid deposition is necessary to determine if the lack of association between ante- and post-mortem measures of amyloid can be resolved [27]. Finally, we did not measure levels of other complements or related proteins, and their levels may further inform baseline and longitudinal differences between the different biochemical cohorts.

## Conclusions

We propose that along with established CSF AD biomarkers, CSF C3 and FH levels will significantly enhance the distinction of different AD stages in clinical practice and trial design, minimize bias in single clinician or consensusbased diagnostic mechanisms, and reduce the biochemical heterogeneity within the existing syndromic categories. Future studies can assess the correlation between peripheral and central complement activation patterns, especially if complement modulating therapies will be considered in AD.

## Additional file

Additional file 1: Table S1. Demographic and biomarker information for cognitive normal subjects and MCI patients with CSF t-Tau/Aβ42 < 0.39 in the ADNI and Emory cohorts. **Table S2.** Main effects from mixed linear modeling of CSF FH and C3 levels in ADNI. In each model, FH or C3 was entered as the dependent variable; age, gender, presence of APOE ε4 allele, diagnosis, Aβ42, t-Tau, p-Tau_181_, gender X age, presence of APOE ε4 allelle X age, and diagnosis X age were entered as fixed factors; and age was also entered as a random factor. Factors with main effect *p* > 0.10 were removed in a step-wise fashion to arrive at final model. See text and Fig. 2 for effects from different diagnostic categories. **Table S3.** Mixed linear modeling of P_AD_-based diagnostic classification and time (in months) on longitudinal memory and executive functions in the Emory validation cohort. A) Among patients initially classified as MCI with longitudinal follow-up (n=44), reclassification using P_AD_ showed differences in absolute executive Z-scores between those reclassified as likely MCI vs. likely mild AD, but no difference in rates of executive function decline or memory functions. B) Among patients initially classified as mild AD with longitudinal follow-up (n=10), only time was associated with longitudinal memory and executive function decline in this underpowered subgroup. (DOCX 21 kb)
