# Supplementary material for: CSF complement 3 and factor H are staging biomarkers in Alzheimer’s disease
Source: Acta Neuropathol Commun. 2016 Feb 17;4:14. doi: 10.1186/s40478-016-0277-8 (PMC4758165; doi:10.1186/s40478-016-0277-8)
Supplement: Additional file 1: Table S1. — Demographic and biomarker information for cognitive normal subjects and MCI patients with CSF t-Tau/Aβ42 < 0.39 in the ADNI and Emory cohorts. Table S2. Main effects from mixed linear modeling of CSF FH and C3 levels in ADNI. In each model, FH or C3 was entered as the dependent variable; age, gender, presence of APOE ε4 allele, diagnosis, Aβ42, t-Tau, p-Tau181, gender X age, presence of APOE ε4 allelle X age, and diagnosis X age were entered as fixed factors; and age was also entered as a random factor. Factors with main effect p > 0.10 were removed in a step-wise fashion to arrive at final model. See text and Fig. 2 for effects from different diagnostic categories. Table S3. Mixed linear modeling of PAD-based diagnostic classification and time (in months) on longitudinal memory and executive functions in the Emory validation cohort. A) Among patients initially classified as MCI with longitudinal follow-up (n=44), reclassification using PAD showed differences in absolute executive Z-scores between those reclassified as likely MCI vs. likely mild AD, but no difference in rates of executive function decline or memory functions. B) Among patients initially classified as mild AD with longitudinal follow-up (n=10), only time was associated with longitudinal memory and executive function decline in this underpowered subgroup. (DOCX 21 kb) [file 40478_2016_277_MOESM1_ESM.docx]

**CSF complement 3 and factor H are staging biomarkers in Alzheimer’s disease**

*Hu, Watts, Tailor, et al.*

**Additional file 1**

**Table S1.** Demographic and biomarker information for cognitive normal subjects and MCI patients with CSF t-Tau/Aβ42 < 0.39 in the ADNI and Emory cohorts.

|  | Normal Cognition | | MCI-Other |
| --- | --- | --- | --- |
|  | ADNI  (n=115) | Emory  (n=25) | ADNI  (n=63) |
| Male (%) | 58 (50%) | 8 (32%) | 48 (76%) |
| Age (S.D.), yr | 66.1 (9.5) | 66.1 (9.5) | 69.0 (6.8) |
| Education (S.D.), yr | 15.7 (2.8) | 17.2 (1.6) | 15.4 (4.2) |
| Having at least one APOE ε4 allele | 28  (24%) | 8/22  (36%) | 16  (25%) |
| CSF  Aβ42 (pg/mL)  t-Tau (pg/mL)  p-Tau_181_ (pg/mL)  FH (pg/mL)  Z-score, log(C3) | 205.9 (54.7)  69.8 (30.4)  24.9 (14.5)  1650 (712)  0.020 (0.873) | 252.2 (113.3)  46.5 (29.6)  27.5 (16.4)  1240 (660)  0.027 (1.037) | 223.1 (44.8)  56.3 (15.3)  19.7 (7.0)  1774 (712)  0.398 (0.930) |

**Table S2**. Main effects from mixed linear modeling of CSF FH and C3 levels in ADNI. In each model, FH or C3 was entered as the dependent variable; age, gender, presence of APOE ε4 allele, diagnosis, Aβ42, t-Tau, p-Tau_181_, gender X age, presence of APOE ε4 allelle X age, and diagnosis X age were entered as fixed factors; and age was also entered as a random factor. Factors with main effect p > 0.10 were removed in a step-wise fashion to arrive at final model. See text and Figure 2 for effects from different diagnostic categories.

CSF C3 levels

|  | F | p |
| --- | --- | --- |
| Intercept | 16.307 | <0.001 |
| Diagnosis | 2.583 | 0.053 |
| Aβ42 | 3.693 | 0.055 |
| Age | 13.986 | <0.001 |

CSF FH levels

|  | F | p |
| --- | --- | --- |
| Intercept | 0.248 | 0.619 |
| Diagnosis | 2.884 | 0.036 |
| Diagnosis x age | 2.610 | 0.051 |
| Aβ42 | 3.044 | 0.082 |
| Age | 12.145 | 0.001 |
| p-Tau_181_ | 12.910 | <0.001 |

**Table S3**. Mixed linear modeling of P_AD_-based diagnostic classification and time (in months) on longitudinal memory and executive functions in the Emory validation cohort. A) Among patients initially classified as MCI with longitudinal follow-up (n=44), reclassification using P_AD_ showed differences in absolute executive Z-scores between those reclassified as likely MCI vs. likely mild AD, but no difference in rates of executive function decline or memory functions. B) Among patients initially classified as mild AD with longitudinal follow-up (n=10), only time was associated with longitudinal memory and executive function decline in this underpowered subgroup.

1. Initial diagnosis of MCI

Longitudinal memory Z-scores

|  | B (95% CI) | p |
| --- | --- | --- |
| **Intercept** | **-2.49 (-3.23, -1.72)** | **<0.001** |
| **Months** | **-0.027 (-0.054, -0.001)** | **0.041** |
| P_AD_  <50% AD  ≥50% AD | Reference  -0.78 (-1.66, 0.09) | 0.078 |
| P_AD_ x Months  <50% AD x Months  ≥50% AD x Months | Reference  -0.009 (-0.039, 0.021) | 0.528 |

Longitudinal executive Z-scores

|  | B (95% CI) | p |
| --- | --- | --- |
| **Intercept** | **-0.99 (-1.24, -0.75)** | **<0.001** |
| Months | -0.066 (-0.324, 0.193) | 0.619 |
| **P_AD_**  **<50% AD**  **≥50% AD** | **Reference**  **-0.48 (-0.77, -0.20)** | **0.001** |
| P_AD_ x Months  <50% AD x Months  ≥50% AD x Months | Reference  -0.040 (-0.340, 0.260) | 0.793 |

1. Initial diagnosis of mild AD

Longitudinal memory Z-scores

|  | B (95% CI) | p |
| --- | --- | --- |
| **Intercept** | **-3.38 (-4.33, -2.44)** | **<0.001** |
| Months | -0.002 (-0.222, 0.217) | 0.982 |
| P_AD_  <50% AD  ≥50% AD | 0.94 (-0.29, 2.16)  Reference | 0.124 |
| P_AD_ x Months  <50% AD x Months  ≥50% AD x Months | 0.002 (-0.282, 0.286)  Reference | 0.988 |

Longitudinal executive Z-scores

|  | B (95% CI) | p |
| --- | --- | --- |
| **Intercept** | **-2.57 (-3.80, -1.34)** | **0.001** |
| **Months** | **-0.030 (-0.045, -0.014)** | **0.001** |
| P_AD_  <50% AD  ≥50% AD | 0.40 (-1.19, 1.99)  Reference | 0.591 |
| **P_AD_ x Months**  **<50% AD x Months**  **≥50% AD x Months** | **0.035 (0.011, 0.059)**  **Reference** | **0.007** |
